# Unusual presentation of an inflamed dentigerous cyst with aggressive features: A case report with management considerations

**DOI:** 10.1016/j.ijscr.2025.111516

**Published:** 2025-06-14

**Authors:** Mhammad Ali, Ali Abbas, Bisan Alshaer, Karam Ahmad, Abdul-Karim Khalil

**Affiliations:** a Al-Andalus University for Medical Science, Faculty of Dentistry, Syria; b Tishreen University Hospital, Department of Oral and Maxillofacial Surgery, Syria; c Al-Andalus University for Medical Sciences, Department of Oral and Maxillofacial Surgery, Syria

**Keywords:** Dentigerous cyst, Odontogenic cysts, Oral and maxillofacial surgery, Case report

## Abstract

**Introduction:**

Dentigerous cysts are the second most common odontogenic cysts, typically asymptomatic and discovered incidentally on radiographs surrounding the crown of impacted teeth. They usually present as well-defined unilocular radiolucencies and exhibit non-inflammatory histological characteristics. In rare instances, they may undergo secondary infection or exhibit aggressive behaviour, posing diagnostic and therapeutic challenges.

**Presentation of case:**

A 64-year-old male patient presented with a progressive mandibular expansion and associated pain as a chief complaint. The oral examination revealed a swelling in the anterior portion of mandible with buccal plate expansion. The radiographic and histopathologic features were consistent with the diagnosis of dentigerous cyst. Consequently, the lesion was surgically removed, and no clinical or radiological recurrence was detected during 12 months post-operative follow-up.

**Discussion:**

While previous reports of dentigerous cysts typically describe a benign course with well-defined radiolucent borders, this case presents an aggressive variant with inflammatory changes. Furthermore, while dentigerous cysts are commonly associated with impacted teeth and remain asymptomatic, our case suggests a potential association between lesion size, patient age, and chronic inflammatory response as possible contributors to its aggressive behaviour.

**Conclusions:**

This case highlights a rare occurrence of aggressive dentigerous cyst with inflammation and cortical perforation. It emphasizes the effectiveness of conservative surgical management and suggests further research on the link between lesion size, age, and unidentified inflammatory factors.

## Highlights

• An atypical presentation of an inflamed dentigerous cyst mimicking an aggressive jaw lesion.
• Findings from this DC case suggest a potential link between cyst size, advanced age, and inflammatory changes.
• This case highlights key considerations in identifying and managing atypical forms of dentigerous cysts.
• Surgical enucleation effectively managed an extensive dentigerous cyst, yielding satisfactory clinical outcomes.

## 1 Introduction

Dentigerous cysts are the most common developmental odontogenic cysts, accounting for approximately 25 % of all odontogenic cysts of the jaws. They are frequently noted as an incidental finding on radiographs because a majority of these cysts are asymptomatic and are most commonly associated with impacted mandibular third molars and permanent maxillary canines [1].

DCs have been studied over time in great detail, providing some insight into how they originate, the nature of their biology and the manifestations of the same in a general populace [2]. It develops in 2 ways: by accumulation of fluid between the reduced enamel epithelium (REE) and the crown or between the layers of the REE [3]. The occurrence of dentigerous cysts has been estimated at 1.44 per 100 unerupted teeth. In addition, the relative risk for individual teeth to develop dentigerous cysts varies considerably [3].

A dentigerous cyst presents as a well-defined radiolucent entity surrounding the crown of an impacted tooth. The border of the cyst is continuous with the cemento-enamel junction of the impacted tooth [4]. Large cysts may cause cortical bone expansion. Moreover, DCs can rarely show a multilocular feature in the panoramic radiography. This is probably due to the cyst growth in areas of different bone densities [5]. Histologically, DCs are represented by a cavity lined by the non-keratinizing thin epithelium without rete pegs. Their wall is usually fibrous and devoid of inflammatory cells [6]. Although dentigerous cysts are typically asymptomatic and benign, rare cases can present with unusual radiographic and clinical features. This case highlights an inflamed dentigerous cyst with multilocular radiolucency, cortical perforation, and an aggressive clinical course, which adds to the spectrum of known presentations and challenges conventional understanding of the lesion's behaviour. The work has been reported in line with the SCARE 2025 criteria [7].

## 2 Presentation of case

A 64-year-old male patient was referred to the department of oral and maxillofacial surgery in January 2024 with a chief complaint of asymptomatic growth localized in the anterior portion of mandible. The patient reported that it was first appeared spontaneously 10 months prior before it invasively enlarged in size and developed into painful swelling. No significant or serious events were reported in the patient's medical history and his oral hygiene was assessed as being in good condition generally. Overall, the patient's medical history is unremarkable for chronic diseases, previous surgeries or hospitalizations, and exhibits no significant systemic health concerns.

**Fig. 1:**
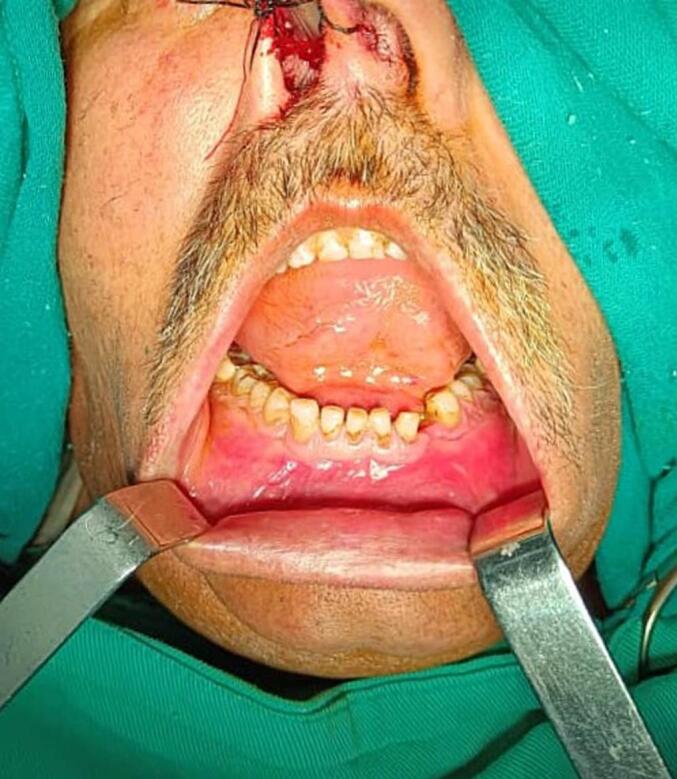
Pre-operative figure shows the swelling and buccal expansion of cortical plate.

The extraoral examination revealed a non-tender firm swelling with buccal bulge over the chin occupying the anterior portion of mandible. Regional lymph nodes were not palpable and facial asymmetry was in normal limits. The egg-shell cracking was obviously felt in palpation which identifying the thinness of the overlying cortex. Intraoral examination features showed clearly the expansion of buccal cortical plate in premolars region, and the lingual cortex was seemed to be intact. A comprehensive dental evaluation was performed for all teeth associated with the lesion, spanning from tooth 37 to 47. Clinically and radiographically, these teeth exhibited no signs of displacement, mobility, or root resorption. All involved teeth were assessed for vitality test and responded positively, except for tooth 37, which had undergone prior endodontic treatment. Tooth 33 was fully impacted and located centrally within the cystic cavity, demonstrating no signs of eruption. The adjacent roots appeared intact and unaffected by the cystic process and there were no periapical lesions associated with these teeth. Overlying oral mucosa of the swelling was apparently normal with no ulceration or drainage related [Fig. 1].

**Fig. 2:**
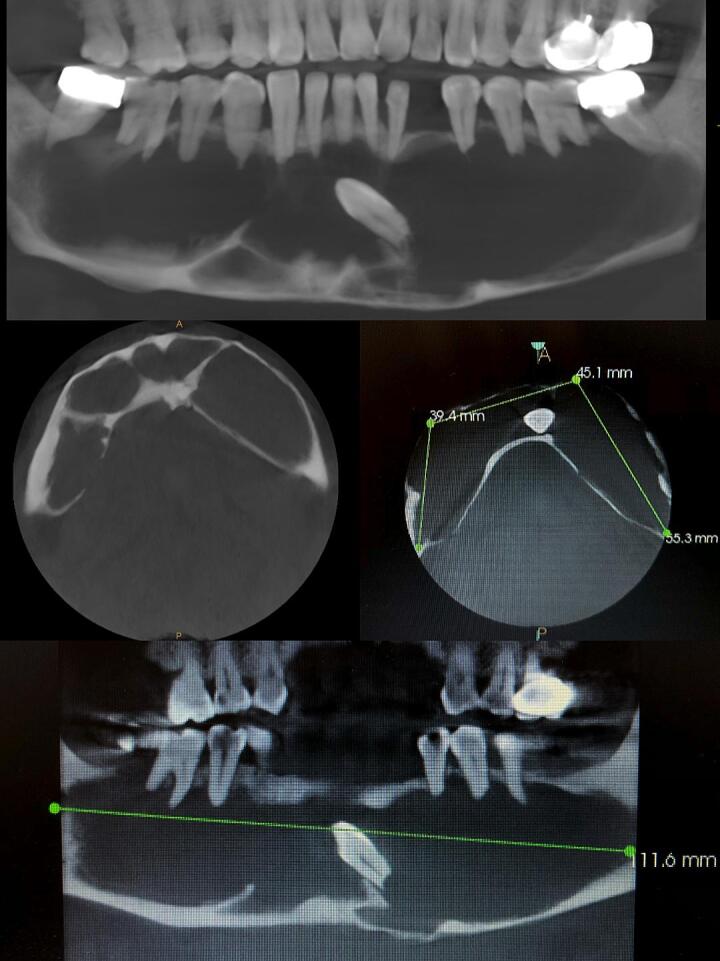
CT images reveal the invasive size and multi-chamber appearance of the lesion.

OPG displayed an extensive well-defined radiolucency involving the entire body of mandible from tooth 37 to 47, measuring approximately 10 × 4 cm and the tooth 33 was completely embedded within the lesion. Because of the size, and site of the lesion in the present case, CT imaging was necessary for the evaluation of cortical plate thickness, and relationship of the lesion with the adjacent structures. Computed tomography revealed extensive osteodestruction divided into four separated chambers and outspread expansion of the buccal side of mandible with perforation of the cortical plates on the same side [Fig. 2]. All the teeth related to the lesion were vital. Accordingly, dentigerous cyst, odontogenic keratocyst and unicystic ameloblastoma were considered for the differential diagnosis.

**Fig. 3:**
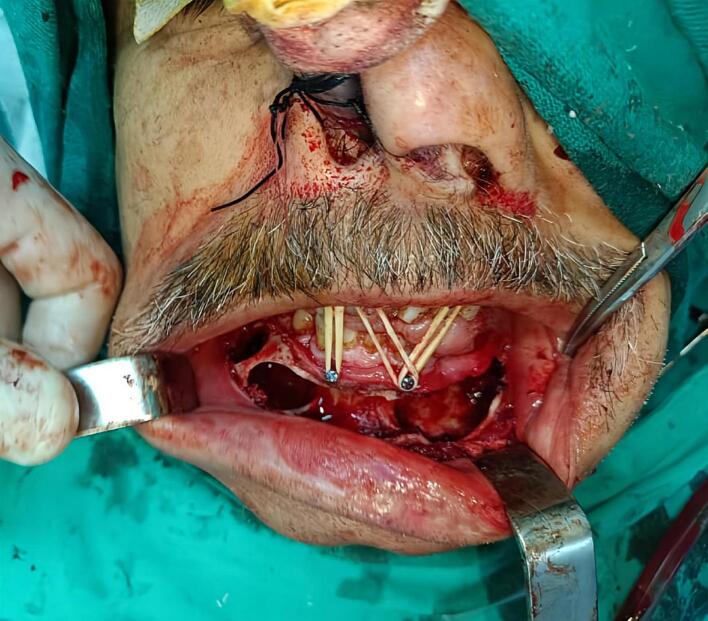
Intraoperative figure showing the surgical windows and enucleated cavity using Partsch II technique.

Depending on clinical and radiographic findings of the case we presented, surgical intervention using enucleation was performed as a treatment procedure to remove the lesion by creating three surgical bone windows through extraction the involved tooth 33. Cystic lumen was thoroughly irrigated with normal saline and betadine solution, followed by the placement of an iodoform-glycerin gauze dressing into the cystic cavity [Fig. 3]. An excisional biopsy was conducted, and the obtained tissue fragments were sent for histopathological examination. The patient was prescribed antibiotics and NSAIDs and scheduled for regular follow-ups to change the dressing.

**Fig. 4:**
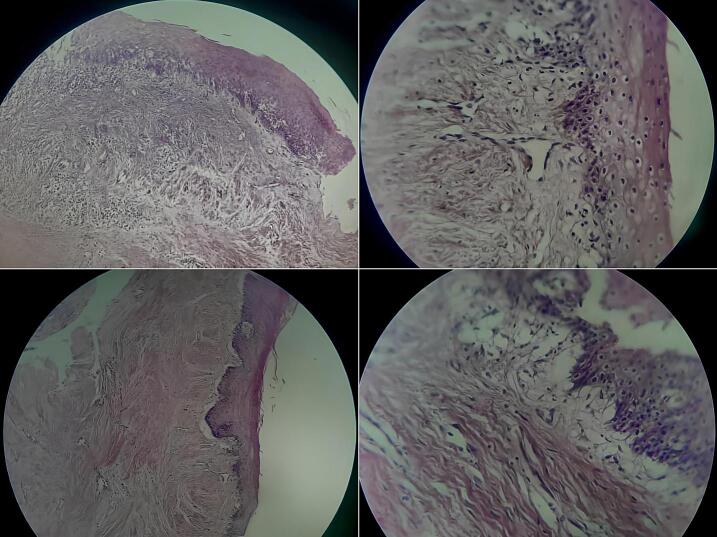
Microscopic features show cystic cavity lined by non-keratinized, stratified epithelium, inflammatory infiltration of chronic and acute cells with cholesterol crystals.

**Fig. 5:**
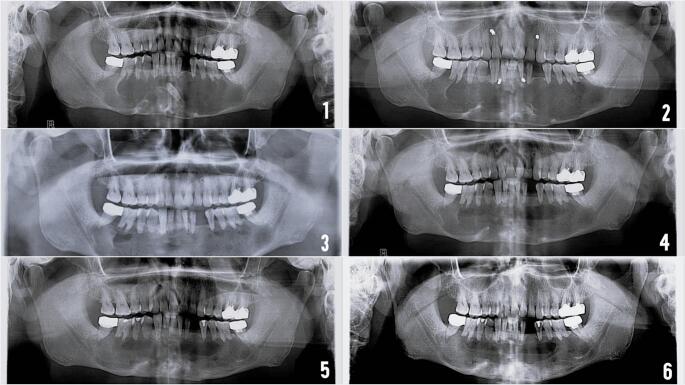
(1) Pre-operative OPG showing a large radiolucent lesion occupying the mandibular body. (2) Immediate post-operative OPG following surgical enucleation. (3–6) Follow-up OPGs at 3, 6, 9, and 12 months, demonstrating progressive bone regeneration.

Microscopical examination demonstrated abundant heavy inflammatory infiltration of chronic and acute cells, along with cholesterol crystals, granulation tissue, and fibrous tissue. Focal areas of residual non-keratinizing squamous epithelium were observed. No features characteristic of odontogenic keratocyst, such as a parakeratinized epithelial lining with palisaded basal cells, nor the plexiform or follicular patterns typical of unicystic ameloblastoma were observed. Additionally, no evidence of cellular atypia or dysplasia was detected, ruling out malignancy and confirming the diagnosis of an inflamed dentigerous cyst [Fig. 4]. Subsequently, the patient remained under observation over 12 months postoperatively. No signs of infection or recurrence were detected during the follow-up period. Sensory function of the lower lip remained intact, and regular follow-up assessments confirmed that all erupted teeth within this region maintained positive vitality responses, with no signs of mobility, root resorption, or periapical pathology. The occlusal relationship and function remained stable, and the overlying soft tissues healed uneventfully [Fig. 5]. The patient reported significant improvement in symptoms following surgical intervention and expressed satisfaction with the treatment outcome and follow-up care. He noted relief from discomfort and confidence in the management approach adopted by the clinical team.

## 3 Discussion

This case report describes a rare clinical presentation and radiographic appearance of dentigerous cyst affecting both the anterior and posterior regions of the mandible, associated with an atypical impaction of permanent mandibular canine. Dentigerous cyst is the most common type of odontogenic cysts, developing around the crown of an unerupted tooth, seemingly without an inflammatory stimulus [5]. It generally presents the second to third decades of life, and is less commonly observed in older individuals. The majority of DC cases involve the mandibular third molars, followed in order of occurrence by the maxillary permanent canines, mandibular second premolars, and maxillary third molars [2,3]. The presence of a large, inflamed dentigerous cyst in a 64-year-old patient is rare and diverges from the typical demographic and anatomical presentation, as most cases are smaller and limited to posterior mandible, and occur almost exclusively in younger individuals [8,9]. The case we present is an inflamed type of massive dentigerous cyst in a 64-year-old male patient, which appeared spontaneously and progressively enlarged, accompanied by swelling and pain as main symptoms.

Radiographically, dentigerous cysts typically present as solitary, unilocular radiolucent areas (89.1 %) with well-defined borders. Multiple cysts of DC in a single jaw are rarely occur and, in such cases, is often associated with syndromes such as Maroteaux-Lamy syndrome and cleidocranial dysplasia [2,10]. However, the OPG in our case revealed a wide radiolucency occupying the entire body of the mandible from tooth 37 to 47. Interestingly, CT imaging revealed a multilocular appearance with four distinct chambers and extensive expansion of the buccal cortical plates. However, no syndromic or systemic abnormalities were identified in the patient's medical history, and notably, it is unusual for a dentigerous cyst to extend across multiple quadrants of the jaw or crossing the midline [11]. Radiographic features demonstrated the circumferential-type of DC, completely encircling both the crown and root of the associated canine, as classified by Shear et al. [10] These findings suggest that lesion size, advanced patient age, or prolonged subclinical irritation may have contributed to the lesion's inflammatory transformation and expansive nature. This combination of atypical features posed both diagnostic and therapeutic challenges. Clinically and radiographically, the lesion raised suspicion for more aggressive entities, requiring comprehensive evaluation through CT imaging and histopathological confirmation to establish a definitive diagnosis.

Various studies suggested two different types of dentigerous cyst, developmental and inflammatory in origin. Inflammatory dentigerous cyst cases reported in the literature have been described as arising from a necrotic overlying deciduous tooth, leading to the accumulation of inflammatory exudates that involve the follicle of the unerupted permanent successor, mostly premolars, ensued with resultant dentigerous cyst formation [3,12]. Nevertheless, retrospective study has shown that the mandibular ramus, particularly in association with an unerupted third molar, is the most common site for infection in dentigerous cyst cases [13]. Regarding the presented case, histopathological examination illustrated a significant inflammatory infiltrate composed of both chronic and acute inflammatory cells. Focal epithelial hyperplasia was not observed, ruling out the possible cause of secondary infection as described [12]. No previous notable oral hygiene-related issues were reported, and all the teeth were intact. The lesion was associated with the impacted tooth 33, with no correlation to or presence of deciduous teeth.

Regarding treatment modalities, factors such as lesion size, patient age, aggressive nature, and involvement of vital structures are considered in determining the appropriate treatment approach. It is crucial to consider the thinness of cortical borders and perforation when selecting the treatment approach that ensures bone stability and reduces the risk of pathological fractures. In addition, the risk of squamous cell carcinoma or malignant transformation arising from a dentigerous cyst increases with age, with an average reported occurrence at 60.8 years. They described that the pluripotent nature of the odontogenic epithelium around the tooth determines its increased chance to become cystic or malignant in the future [14]. Two surgical methods have been suggested for the management of dentigerous cysts. Marsupialization (Partsch I technique) is primarily considered the treatment of choice for inflammatory dentigerous cysts and is recommended for preserving the associated tooth and adjacent anatomical structures, particularly in cases of mixed dentition [2,15]. The other surgical method, complete enucleation (Partsch II technique), is recommended for cases in adult patients where the cyst is associated with an impacted permanent tooth. This approach involves the complete surgical removal of the cystic lining along with the associated tooth, aiming to eliminate the lesion in its entirety and reduce the risk of recurrence [2]. Conservative enucleation remains a preferred treatment option when feasible, allowing complete removal while preserving surrounding tissues.

Recent literature suggests that in cases involving large cystic lesions with cortical thinning, a two-stage surgical approach involving initial marsupialization followed by delayed enucleation may reduce the risk of mandibular fracture and promote bone regeneration [15]. This approach is particularly advised when preserving mandibular continuity is a priority. While we opted for primary enucleation due to lesion accessibility and patient age, future protocols might benefit from a combined strategy in younger patients or when higher fracture risk is anticipated.

Accordingly, the surgical procedure using the Partsch II technique was carried out as the treatment method, in accordance with the recommendations of the American Association of Oral and Maxillofacial Surgeons (AAOMS) guideline, and considering the clinical risk factors that could lead to high rate of morbidity and recurrence if the first method was applied. Enucleation is especially indicated when preservation of the tooth is not feasible and when the lesion shows radiographic or histopathologic signs of aggressiveness [2]. This method proved effective in managing the lesion's size and complexity, preserving the neurovascular integrity and adjacent teeth, and promoting uneventful healing through secondary intention supported by medicated gauze packing. Follow-up for 12 months postoperatively was recommended in literature, showing satisfactory results with favourable healing outcomes, including progressive reduction of the radiolucency.

## 4 Conclusions

This case presents a rare dentigerous cyst with aggressive behaviour, including cortical perforation and inflammatory changes, which are atypical for this lesion type. It underscores the importance of considering conservative surgical approaches in dentigerous cyst cases, even when complicated by such extensive structural involvement, to preserve mandibular integrity and minimize morbidity. It also emphasizes the importance of further studies to explore potential correlations between cyst size and advanced age, as well as unknown factors contributing to inflammatory changes in dentigerous cysts.

## Informed consent

Written informed consent was obtained from the patient for publication of this case report and accompanying images. A copy of the written consent is available for review by the Editor-in-Chief of this journal on request.

## Ethical approval

Ethical approval is waived at our institution, and this study was exempt from ethical approval at our institution, as this paper reports a single case that emerged during normal surgical case report.

## Funding

No source of funding.

## Author contribution

MA: Writing and drafting the article, Critical revision.

AA, BA: Data Collection and Preparation.

AKh, KA: Supervision.

All authors provided final approval of the version to be submitted.

## Guarantor

Abdul-Karim Khalil DDS, OMFS, PhD.

## Research registration number

N/A.

## Conflict of interest statement

None.

## References

[1] Daley T.D., Wysocki G.P., Pringle G.A. (1994). Relative incidence of odontogenic tumors and oral and jaw cysts in a Canadian population. Oral Surg. Oral Med. Oral Pathol..

[2] Joshi M., Samuel S., Abraham A., Deepak D.M. (2021). Management of Dentigerous Cysts - a review. J. Evol. Med. Dent. Sci..

[3] Narang R.S., Manchanda A.S., Arora P., Randhawa K. (2012). Dentigerous cyst of inflammatory origin-a diagnostic dilemma. Ann. Diagn. Pathol..

[4] White S.C., Pharoah M.J. (2008). https://books.google.co.id/books?id=_GWQ13qK_-kC.

[5] Scholl R.J., Kellett H.M., Neumann D.P., Lurie A.G. (1999). Cysts and cystic lesions of the mandible: clinical and radiologic-histopathologic review. Radiographics.

[6] NEVILLE B.W. (2009). https://www.worldcat.org/title/834142726.

[7] Kerwan A., Al-Jabir A., Mathew G., Sohrabi C., Rashid R., Franchi T., Nicola M., Agha M., Agha R. (2025). Revised surgical CAse REport (SCARE) guideline: an update for the age of artificial intelligence. Prem. J. Sci..

[8] Ghandour L., Bahmad H.F., Bou-Assi S. (2018). Conservative treatment of Dentigerous cyst by marsupialization in a young female patient: a case report and review of the literature. Case Rep. Dent..

[9] Demiriz L., Misir A.F., Gorur D.I. (2015). Dentigerous cyst in a young child. Eur. J. Dent..

[10] Shear M., Speight P. (2008).

[11] Motamedi M.H.K., Talesh K.T. (2005). Management of extensive dentigerous cysts. Br. Dent. J..

[12] Benn A., Altini M. (1996). Dentigerous cysts of inflammatory origin. A clinicopathologic study. Oral Surg. Oral Med. Oral Pathol. Oral Radiol. Endod..

[13] Smith J.L., Kellman R.M. (2005). Dentigerous cysts presenting as head and neck infections. Otolaryngol. Head Neck Surg..

[14] Manganaro A.M., Cross S.E., Startzell J.M. (1997). Carcinoma arising in a dentigerous cyst with neck metastasis. Head Neck.

[15] Van Phan T., Phan D.G., Phan H.M., Nguyen H.M. (2024). Marsupialization followed by enucleation of a large maxillary dentigerous cyst in a young child: a case report and literature review. Int. J. Surg. Case Rep..

